# Supplementary material for: Three previously characterized resistances to yellow rust are encoded by a single locus Wtk1
Source: J Exp Bot. 2020 Jan 14;71(9):2561–72. doi: 10.1093/jxb/eraa020 (PMC7210774; doi:10.1093/jxb/eraa020)
Supplement: eraa020_suppl_supplementary_tables_S1_S3_figures_S1_S2 [file eraa020_suppl_supplementary_tables_s1_s3_figures_s1_s2.pdf]

Supplementary Information for:

Three previously characterized resistances to yellow rust are encoded by a single locus *Wtk1*

Valentyna Klymiuk[1,2,3], Andrii Fatiukha[1,2], Dina Raats[1,2], Valeria Bocharova[1,2], Lin Huang[1,2], Lihua Feng[1,2], Samidha Jaiwar[1], Curtis Pozniak[3], Gitta Coaker[4], Jorge Dubcovsky[5,6], Tzion Fahima[1,2*]

Table S1. A list of SSR, CAPS and KASP markers used in the current study.

Name of marker	Type of marker	Primer sequence	Reference
gwm413	SSR	TGCTTGTCTAGATTGCTTGGG<br>GATCGTCTCGTCCTTGGCA	Röder *et al.* (1998)
wmc406	SSR	TATGAGGGTCGGATCAATACAA<br>CGAGTTTACTGCAAACAAATGG	Somers *et al.* (2004)
barc8	SSR	GCGGGAATCATGCATAGGAAAACAGAA<br>GCGGGGGCGAAACATACACATAAAAACA	Song *et al.* (2005)
*uhw290**	CAPS	ATAGCAGGATCGCAGCAAAA<br>GAGGTATAACAATGTGATCGTTCTG	Current study
uhw250	EST	CTGCTCACTTTTTGCCTGTG<br>AAAAGTTGTTGCTCTGCTTTT	Klymiuk *et al.* 2018
uhw256	EST	GTTACCCTCCACAGCAAGGT<br>GCGCATTACTTCCACTTCTTG	Klymiuk *et al.* 2018
RAC875_c826_839	KASP	ACGAAGGTTCTGTTTTCACCA<br>ACGAAGGTTCTGTTTTCACCG<br>TCTTCTTGCTCAAAGGTAAGAGT	Klymiuk *et al.* 2018
uhw255	CAPS	GATGCTCTGCACATGTGTTATG<br>GCAGCTCCAGCTTATTCGTC	Klymiuk *et al.* 2018
uhw264	CAPS	GGTCTCTTGCAACATACAGTAACAA<br>GAGTGGTAGTCTAGTAGAGGTTGGTG	Klymiuk *et al.* 2018
uhw267	CAPS	TGGTAATCAAGTTTCACATTGTTCA<br>GGAAGGACACCTTTCGGTATT	Klymiuk *et al.* 2018
uhw297	CAPS	CAGATGACCAACCAAAAGCA<br>GTCATATTGGTGCCCAGTGA	Klymiuk *et al.* 2018
uhw292	dominant	GACTTTCTTCCCTCGGGACT<br>CTCGCACGCCTATAAAAGGA	Klymiuk *et al.* 2018
uhw300	dominant	CCGTGTCAGCCACCTACAAT<br>GCACTCTACCACCGAACACA	Klymiuk *et al.* 2018
uhw301	dominant	GTAGTGGCTCGTTCGGTGAT<br>TTTCGCATCCCACCCTACTG	Klymiuk *et al.* 2018
uhw296	CAPS	CAACCGTGCCTCCAAACA<br>CGGGTGTTGTCCGTTGAG	Klymiuk *et al.* 2018
uhw302	dominant	CATCCATTCCTCCGACAAGT<br>CACTGCAATGCAAAAATGCT	Klymiuk *et al.* 2018
uhw276	CAPS	TCTGTGATGCCTGTGATGGT<br>AAAGTTTGGGATTTGGCAAT	Klymiuk *et al.* 2018
uhw273	CAPS	GGTGACGGCGAGTGTACG<br>GACGCAATTGTCCGCTGT	Klymiuk *et al.* 2018
uhw274	CAPS	AAGCTCCGCTGCAATGAC<br>ACCTGACATCCTCGAACCAC	Klymiuk *et al.* 2018
uhw259	CAPS	CTGTATTCTAATGCAGATTAGCTGTT<br>CACGCATAATTTTGTCCACAC	Klymiuk *et al.* 2018
BS00022902_51	KASP	ATGTGCGGCAGGAGAAGA<br>ATGTGCGGCAGGAGAAGG<br>ATACTCTTCACGGTCGTCTTC	Klymiuk *et al.* 2018
gwm273	SSR	ATTGGACGGACAGATGCTTT<br>AGCAGTGAGGAAGGGGATC	Röder *et al.* (1998)

* Temperature of elongation 59°C; Enzyme *Aci*I; Fragment sizes: *T. dicoccoides* H52 = 266 bp, 148 bp; *T. durum* cv. Langdon = 414 bp.

Table S2. A list of KASP markers from the *Yr15* region developed based on SNPs from the wheat 15K SNP array.

Name of marker	Primer A	Primer B	Primer Common
RFL_Contig2160_617	cgtgtgaatgtgtactctacca	cgtgtgaatgtgtactctaccg	ggacccagaatgcccagtg
IACX502	cacgcctaacaaaacatatccatt	cacgcctaacaaaacatatccatc	tgggagttcttatttaatctttcgg
Ra_c16879_977	cagagcaaaagccatcaatctta	cagagcaaaagccatcaatcttc	tctgagaaagatgccagaacg
BS00087784_51	gagctgacagatgggggt	gagctgacagatgggggc	ccatgacatcagaaaagttgatgat
Excalibur_c17202_1833	gttagagcccaattttcaagcta	gttagagcccaattttcaagctg	aggagcttatattgctatgtacagt
wsnp_Ku_c4911_8795151	gttcatcaactgttgagctgtt	gttcatcaactgttgagctgtc	gctgctgtagtttctgattgtg
wsnp_Ex_c2111_3963161	agcagcatacgattaactcagtt	agcagcatacgattaactcagtc	ctgttggcgcagaagctg
RAC875_c79370_378	tggaactgatggtgtccagt	tggaactgatggtgtccagc	gcggcatcaacttccccg

Table S3. Molecular characterization of the *yr15*[#], *yrG303* and *yrH52* EMS mutants

Domain	Base substitution*	Effect on amino acid†	Gene	Mutant No.	Name of mutant
KinI	G 160 A	G 54 S	*yr15*	EMS1	Suncea+*yr15*-L18
KinI	G 161 A	G 54 D	*yrH52*	EMS14	M52-4
KinI	G 340 A	E 114 K	*yr15*	EMS2	Suncea+*yr15*-L89
KinI	G 445 A	A 149 T	*yrG303*	EMS11	M2298-410-16
KinI	G 445 A	A 149 T	*yrH52*	EMS15	M52-9
KinI	G 448 A	E 150 K	*yr15*	EMS3	B9+*yr15*-L1351
KinI	C 482 T	P 161 L	*yr15*	EMS4	Avocet+*yr15*-1
KinI	C 509 T	A 170 V	*yrH52*	EMS16	M52-14
KinI	C 602 T	T 201 I	*yrH52*	EMS17	M52-18
KinI	C 632 T	P 211 L	*yr15*	EMS5	Avocet+*yr15*-L90
KinI	G 686 A	G 229 E	*yrH52*	EMS18	M52-2
KinI	G 705 A	M 235 I	*yrG303*	EMS12	A95-126
KinII	G 2922 A	A 460 T	*yr15*	EMS6	Avocet+*yr15*-13
KinII	G 2922 A	A 460 T	*yrH52*	EMS19	M52-8
KinII	G 3114 A	D 524 N	*yr15*	EMS7	Excalibur+*yr15*-6L306
KinII	C 3229 T	T 562 I	*yr15*	EMS8	Avocet+*yr15*-L72
KinII	G 3281 A	W 581 *	*yrG303*	EMS13	M2298-767-16
KinII	C 3315 T	P 591 S	*yr15*	EMS9	B9+*yr15*-LF
KinII	G 3469 A	V 614 M	*yr15*	EMS10	Excalibur+*yr15*-L137

[#]Full description of *yr15* mutants is provided in Klymiuk *et al.* (2018).

[*]The first letter indicates the wild-type nucleotide, the number indicates its position relative to the ATG start codon, and the last letter shows the mutant nucleotide. The complete WTK1 coding regions of the above 19 mutants were sequenced; no additional mutations were detected.

[†]The first letter indicates the wild-type amino acid, the number indicates its position relative to the start methionine, and the last letter shows the mutant amino acid.

#RIL	*barc8*	*IACX502*	*Ra_c16879_977*	*uhw290*	*Excalibur_c17202_1833*	*wsnp_Ku_c4911_8795151*	*wsnp_Ex_c2111_3963161*	*RAC875_c826_839*	*uhw255*	*uhw264*	*RAC875_c79370_378*	*uhw297*	*uhw292*	*uhw300*	*YrG303* seedling	*YrG303* adult	*uhw301*	*uhw296*	*uhw302*	*uhw276*	*uhw273*	*uhw274*	*uhw259*	*BS00022902_51*	*gwm273*
2_6_38_3	B	B	B	B	B	B	B	B	B	B	B	B	B	B	B	B	B	B	B	B	B	B	B	B	A
1_1_82_1	B	B	B	B	B	B	B	B	B	B	B	B	B	B	B	B	B	B	B	B	B	B	B	B	A
1_1_84_5	B	B	B	B	B	B	B	B	B	B	B	B	B	B	B	B	B	B	B	B	B	B	B	A	A
2_2_92_10	B	B	B	B	B	B	B	B	B	B	B	B	B	B	B	B	B	B	B	B	B	B	B	A	A
2_6_50_1	B	B	B	B	B	B	B	B	B	B	B	B	B	B	B	B	B	B	B	B	B	B	A	A	A
2_3_51_3	B	B	B	B	B	B	B	B	B	B	B	B	B	B	B	B	B	B	A	A	A	A	A	A	A
2_4_25_3	B	B	B	B	B	B	B	B	B	B	B	B	B	B	B	B	B	A	A	A	A	A	A	A	A
1_3_19_4	B	B	B	B	B	B	B	B	B	B	B	A	A	A	A	A	A	A	A	A	A	A	A	A	A
2_1_12_10	B	B	B	B	B	B	B	B	B	B	A	A	A	A	A	A	A	A	A	A	A	A	A	A	A
1_2_66_10	B	B	B	B	B	B	B	B	B	B	A	A	A	A	A	A	A	A	A	A	A	A	A	A	A
2_4_23_5	B	B	B	B	B	B	B	B	A	A	A	A	A	A	A	A	A	A	A	A	A	A	A	A	A
2_1_54_2	B	B	B	B	B	A	A	A	A	A	A	A	A	A	A	A	A	A	A	A	A	A	A	A	A
2_3_32_4	B	B	B	B	A	A	A	A	A	A	A	A	A	A	A	A	A	A	A	A	A	A	A	A	A
1_2_70_1	B	B	B	B	A	A	A	A	A	A	A	A	A	A	A	A	A	A	A	A	A	A	A	A	A
3_2_74_4	B	B	B	A	A	A	A	A	A	A	A	A	A	A	A	A	A	A	A	A	A	A	A	A	A
2_1_64_1	B	B	A	A	A	A	A	A	A	A	A	A	A	A	A	A	A	A	A	A	A	A	A	A	A
2_4_88_4	B	A	A	A	A	A	A	A	A	A	A	A	A	A	B	A	A	A	A	A	A	A	A	A	A
3_2_64_2	B	A	A	A	A	A	A	A	A	A	A	A	A	A	A	A	A	A	A	A	A	A	A	A	A
2_2_60_2	B	A	A	A	A	A	A	A	A	A	A	A	A	A	A	A	A	A	A	A	A	A	A	A	A
1_1_66_4	A	A	A	A	A	A	A	A	A	A	A	A	A	A	B	A	A	A	A	A	A	A	A	A	B
2_7_4_3	A	A	A	A	A	A	A	A	A	A	A	A	A	A	A	A	A	A	A	A	A	A	A	A	B
3_3_48_3	A	A	A	A	A	A	A	A	A	A	A	A	A	A	A	A	A	A	A	A	A	A	A	A	B
2_1_58_1	A	A	A	A	A	A	A	A	A	A	A	A	A	A	B	A	A	A	A	A	A	A	A	B	B
2_1_59_2	A	A	A	A	A	A	A	A	A	A	A	A	A	A	A	A	A	A	A	A	A	A	A	B	B
1_1_79_1	A	A	A	A	A	A	A	A	A	A	A	A	A	A	A	A	A	A	A	A	A	A	B	B	B
2_5_74_8	A	A	A	A	A	A	A	A	A	A	A	A	A	A	A	A	A	A	A	A	A	A	B	B	B
2_5_80_4	A	A	A	A	A	A	A	A	A	A	A	A	B	B	B	B	B	B	B	B	B	B	B	B	B
1_3_58_9	A	A	A	A	A	A	A	A	A	A	B	B	B	B	B	B	B	B	B	B	B	B	B	B	B
2_4_45_3	A	A	A	A	A	A	A	A	A	B	B	B	B	B	B	B	B	B	B	B	B	B	B	B	B
3_2_5_3	A	A	A	A	A	B	B	B	B	B	B	B	B	B	B	B	B	B	B	B	B	B	B	B	B
2_3_30_2	A	A	A	A	A	B	B	B	B	B	B	B	B	B	B	B	B	B	B	B	B	B	B	B	B
3_3_10_2	A	A	A	A	B	B	B	B	B	B	B	B	B	B	B	B	B	B	B	B	B	B	B	B	B
3_2_26_2	A	A	A	A	B	B	B	B	B	B	B	B	B	B	B	B	B	B	B	B	B	B	B	B	B
3_2_86_2	A	A	A	B	B	B	B	B	B	B	B	B	B	B	B	B	B	B	B	B	B	B	B	B	B
3_2_18_3	A	B	B	B	B	B	B	B	B	B	B	B	B	B	B	B	B	B	B	B	B	B	B	B	B
2_2_7_4	A	B	B	B	B	B	B	B	B	B	B	B	B	B	B	B	B	B	B	B	B	B	B	B	B

Fig. S1. Graphical genotype of selected recombinant lines (RILs) from *YrG303* tetraploid mapping population. Allele A – resistant allele from *T. dicoccoides* acc. G303; allele B – susceptible allele from *T. durum* acc. D447. *YrG303* seedling and *YrG303* adult columns correspond to the phenotypes observed at seedling and adult screens with *Pst* (14 and 21 dpi, respectively). RILs marked in red showed different seedling and adult resistance response.

#RIL	*barc8*	*RFL_Contig2160_617*	*BS00087784_51*	*uhw267*	*uhw290*	*Excalibur_c17202_1833*	*wsnp_Ku_c4911_8795151*	*RAC875_c826_839*	*wsnp_Ex_c2111_3963161*	*uhw255*	*uhw264*	*RAC875_c79370_378*	*uhw297*	*uhw292*	*uhw300*	*YrH52*	*uhw301*	*uhw296*	*uhw259*	*BS00022902_51*	*gwm273*
**V1_31_1**	B	B	B	B	B	B	B	B	B	B	B	B	B	B	B	B	B	B	B	B	A
**V1_60_6**	B	B	B	B	B	B	B	B	B	B	B	B	B	B	B	B	B	B	B	B	A
**GA19_37_12_1**	B	B	B	B	B	B	B	B	B	B	B	B	B	B	B	B	B	B	B	A	A
**GA_10_61_14_2**	B	B	B	B	B	B	B	B	B	B	B	B	A	A	A	A	A	A	A	A	A
**D11_86_6_4**	B	B	B	B	B	B	B	B	B	B	A	A	A	A	A	A	A	A	A	A	A
**GA_22_37_10_3**	B	B	B	B	B	B	B	B	B	A	A	A	A	A	A	A	A	A	A	A	A
**D16_5_11_2**	B	B	B	B	B	B	B	B	B	A	A	A	A	A	A	A	A	A	A	A	A
**V4_5_3**	B	B	B	B	B	B	B	B	B	A	A	A	A	A	A	A	A	A	A	A	A
**V2_90_4**	B	B	B	B	B	B	A	A	A	A	A	A	A	A	A	A	A	A	A	A	A
**D130_4_14_4**	B	B	B	B	B	B	A	A	A	A	A	A	A	A	A	A	A	A	A	A	A
**402_9_20_1_2**	B	B	B	B	B	B	A	A	A	A	A	A	A	A	A	A	A	A	A	A	A
**D128_47_7_4**	B	B	B	B	B	A	A	A	A	A	A	A	A	A	A	A	A	A	A	A	A
**V3_101_2**	B	B	B	B	B	A	A	A	A	A	A	A	A	A	A	A	A	A	A	A	A
**GA_26_27_6_2**	B	B	B	B	B	A	A	A	A	A	A	A	A	A	A	A	A	A	A	A	A
**d131_46_9_1**	B	B	A	A	A	A	A	A	A	A	A	A	A	A	A	A	A	A	A	A	A
**V4_1_2**	B	B	A	A	A	A	A	A	A	A	A	A	A	A	A	A	A	A	A	A	A
**GA26_73_4_1**	B	B	A	A	A	A	A	A	A	A	A	A	A	A	A	A	A	A	A	A	A
**d133_42_16_1**	B	A	A	A	A	A	A	A	A	A	A	A	A	A	A	A	A	A	A	A	A
**d127_9_21_1**	B	A	A	A	A	A	A	A	A	A	A	A	A	A	A	A	A	A	A	A	A
**V4_14_2**	B	A	A	A	A	A	A	A	A	A	A	A	A	A	A	A	A	A	A	A	A
**V1_46_3**	A	A	A	A	A	A	A	A	A	A	A	A	A	A	A	A	A	A	A	A	B
**d16_76_5_1**	A	A	A	A	A	A	A	A	A	A	A	A	A	A	A	A	A	A	A	B	B
**402-18-8-3_2**	A	A	A	A	A	A	A	A	A	A	A	B	B	B	B	B	B	B	B	B	B
**371_8_11_4_2**	A	A	A	A	A	A	B	B	B	B	B	B	B	B	B	B	B	B	B	B	B
**D14_54_1_2**	A	A	A	A	A	A	B	B	B	B	B	B	B	B	B	B	B	B	B	B	B
**GA_18_23_15_2**	A	A	A	A	A	A	B	B	B	B	B	B	B	B	B	B	B	B	B	B	B
**V3_70_3**	A	A	A	A	A	B	B	B	B	B	B	B	B	B	B	B	B	B	B	B	B
**D13_39_5_2**	A	A	A	A	A	B	B	B	B	B	B	B	B	B	B	B	B	B	B	B	B
**GA_17_40_11_2**	A	A	A	A	A	B	B	B	B	B	B	B	B	B	B	B	B	B	B	B	B
**d12_149_3_1**	A	A	B	B	B	B	B	B	B	B	B	B	B	B	B	B	B	B	B	B	B
**d16_57_13_1**	A	A	B	B	B	B	B	B	B	B	B	B	B	B	B	B	B	B	B	B	B
**d12_74_6_1**	A	A	B	B	B	B	B	B	B	B	B	B	B	B	B	B	B	B	B	B	B
**GA19_118_5_1**	A	B	B	B	B	B	B	B	B	B	B	B	B	B	B	B	B	B	B	B	B

Fig. S2. Graphical genotype of selected RILs from *YrH52* mapping population. Allele A – resistant allele from *T. dicoccoides* acc. H52; allele B – susceptible allele from *T. durum* cv. LDN. *YrH52* column corresponds to the phenotypes observed at seedling screen with *Pst* at 14 dpi.

Reference list:

1. Klymiuk V, Yaniv E, Huang L, Raats D, Fatiukha A, Shisheng C, Feng L, Frenkel Z, Krugman T, Lidzbarsky G, Chang W, Jääskeläinen MJ, Schudoma C, Paulin L, Laine P, Bariana H, Sela H, Saleem K, Sørensen CK, Hovmøller MS, Distelfeld A, Chalhoub B, Dubcovsky J, Korol AB, Schulman AH, Fahima T (2018) Cloning of the wheat *Yr15* resistance gene sheds light on the plant tandem kinase-pseudokinase family. Nature Communications 9:3735 (doi:10.1038/s41467-018-06138-9).
2. Röder MS, Korzun V, Wendehake K, Plaschke J, Tixier MH, Leroy P, Ganal MW (1998) A microsatellite map of wheat. Genetics 149:2007-2023.
3. Somers DJ, Isaac P, Edwards K (2004) A high-density microsatellite consensus map for bread wheat (*Triticum aestivum* L.). TAG 109:1105-1114.
4. Song QJ, Shi JR, Singh S, Fickus EW, Costa JM, Lewis J, Gill BS, Ward R, Cregan PB (2005) Development and mapping of microsatellite (SSR) markers in wheat. Theoretical and applied genetics. Theor Appl Gene 110:550-60.
